# Analysis of selected cytokines, NLRP3 inflammasome and α-Klotho protein in patients with heart failure after ICD/CRT-D high-voltage intervention

**DOI:** 10.3389/fphys.2025.1721432

**Published:** 2026-01-07

**Authors:** Jakub Szyller, Mariola Śliwińska-Mossoń, Bruno Hrymniak, Agnieszka Olejnik, Łukasz Kozera, Maciej Zieliński, Waldemar Banasiak, Iwona Bil-Lula, Dariusz Jagielski

**Affiliations:** 1 Faculty of Medicine, Wroclaw University of Science and Technology, Wroclaw, Poland; 2 Lower Silesian Oncology, Pulmonology and Hematology Center, Wroclaw, Poland; 3 Division of Interventional Cardiology, Centre of Heart Diseases, 4th Military Hospital, Wroclaw, Poland; 4 Division of Clinical Chemistry and Laboratory Hematology, Department of Medical Laboratory Diagnostics, Faculty of Pharmacy, Wroclaw Medical University, Wroclaw, Poland; 5 St. Lucas Hospital in Bolesławiec, Bolesławiec, Poland; 6 Department of Cardiology, Centre of Heart Diseases, 4th Military Hospital, Wroclaw, Poland

**Keywords:** arrhythmia, biomarker, cytokines, heart failure, implantablecardioverter-defibrillator, inflammasome, peri-shock-period

## Abstract

**Objectives:**

Inflammatory cytokines contribute to Implantable Cardioverter-Defibrillator/Cardiac Resynchronization Therapy with a Defibrillator (ICD/CRT-D) high-voltage intervention by promoting arrhythmias through direct cardiac effects and indirect systemic changes. The NLRP3 inflammasome can promotes arrhythmias by linking inflammation, oxidative stress, and structural changes. The aim of this study was to assess the inflammatory response in the peri-shock-period in patients with heart failure (HF) and implanted ICD/CRT-D and the initial analysis of the possible role of cytokines, NLRP3 and soluble Klotho protein in peri-shock period and the possibility of triggering arrhythmias.

**Methods:**

The study population consisted of 50 patients with diagnosed HF and implanted ICD/CRT-D devices. Blood samples were drawn up to max. 6 h after appropriate ICD/CRT-D intervention (“intervention group”) or from patients qualified for ICD/CRT-D device replacement (ERI status) and with no intervention min in the previous 3 months (“control group”). Serum concentration of TNF-α, IL-1β, IL-6, IL-10, IL-17, NLRP3 inflammasome, soluble Klotho protein and FGF-23 complete blood count, were determined in all individuals.

**Results:**

IL-6 and IL-10 were higher after ICD/CRT-D appropriate intervention (3.3 pg/mL, 95% CI: 2.7–3.7 vs. 4.6 pg/mL, 95% CI: 2.9–14.5, p = 0.0399 and mean 7.8 pg/mL, 95% CI: 7.1–8.5 vs. 9.0 pg/mL, 95% CI: 8.1–9.9, p = 0.0321, respectively). The group was characterized by a higher number of white blood cells (WBC, 6.8 × 10^3^/µL, 95% CI: 6.0–7.6 vs. 9.4 × 10^3^/µL, 95% CI: 8.1–10.7, p = 0.0017), neutrophils (NEUTs, 4.1 × 10^3^/µL, 95% CI: 3.4–4.8 vs. 6.5 × 10^3^/µL, 95% CI: 5.3–7.7, p = 0.0007) and lower number of eosinophils (EOSs, 0.11 × 10^3^/µL, 95% CI: 0.10–0.16 vs. 0.03 × 10^3^/µL, 95% CI: 0.01–0.08, p = 0.0013). Serum concentration of soluble Klotho protein was significantly higher after device intervention (557.5 pg/mL, 95% CI: 495.5–619.5 vs. 895.2 pg/mL, 95% CI: 744.7–1,046.0, p < 0.0001), with no change in FGF-23 levels.

**Conclusion:**

In the peri-shock period, increased IL-6 and IL-10 serum concentrations and changes in 5-diff blood count (increased neutrophils and decreased eosinophils) are observed, which may be associated with a higher risk of ventricular arrhythmia in HF patients. A significant increase in α-Klotho protein concentration, should be taken into account in the development of future diagnostic methods and indicates an important protective role in the inflammatory process.

## 1 Introduction

Heart failure (HF) remains the leading cause of death worldwide (Bozkurt et al., 2023; Groenewegen et al., 2020). Each type of HF (HF with reduced (HFrEF), mildly reduced (HFmrEF) or with preserved ejection fraction (HFpEF)) is consistently associated with oxidative stress and both local and systemic activation of inflammatory signaling cascades (Dick and Epelman, 2016). Inflammation is a major pathophysiological contributor of HF and accepted cause of unfavorable remodeling after heart muscle damage (Reina-Couto et al., 2021; Westman et al., 2016). It has also been found that in patients with myocarditis or inflammatory cardiomyopathy, inflammation is the main cause of HF (Trachtenberg and Hare, 2017). Nevertheless, the inflammatory processes in HF are extremely complex, multidirectional and require further detailed research (Montuoro et al., 2025; Taylor et al., 2025). Inappropriate targeting of physiological inflammation (which may be required e.g. for reparative activities), can lead to deleterious results. Early studies focused on a single target (e.g., neutralization of the effects of a specific cytokine like TNF-α with etanercept) did not yield good results, which may be dose dependent (Mann et al., 2004). On the other hand, the study with the anti-IL1β monoclonal antibody canakinumab had favorable effects and showed that proper anti-inflammatory action can have positive effects (Ridker et al., 2017) which indicates the need to investigate multiple mechanisms. High mortality in patients with HF is a result of hemodynamic failure, but can also be due to heart electrical disturbances and life-threatening ventricular arrhythmias (VAs) induced by cytokines (Breitenstein and Steffel, 2019).

The electrophysiological mechanisms leading cardiac rhythm disorders have been extensively studied for many years, but the electroimmunological mechanisms remains unclear. Inflammatory cytokines (such as TNF-α, IL-6, IL-1β, IL-17) are involved in the pathogenesis of malignant VAs (ventricular tachycardia (VT) and ventricular fibrillation (VF)), but are largely overlooked in the management of heart rhythm disorders (Lazzerini et al., 2022; Lazzerini et al., 2023; Obeagu, 2025). Moreover, atrial fibrillation (AF) have the best known association with pro-inflammatory cytokines (Niskala et al., 2024; Scott et al., 2019). Also post-operative atrial fibrillation (POAF) has determined correlation with inflammatory processes associated with elevated TNF-α and IL-6 levels (Watt et al., 2021; Zaman et al., 2016). Inflammation process may promote arrhythmias by the development of e.g. ischemic heart disease and cardiac fibrosis or directly by affecting cardiac electrophysiology - especially connexin-43 (Cx-43) downregulation or QT-interval elongation. In the direct electrophysiological mechanism, inflammatory cytokines (e.g. TNF-α, IL-1β, IL-6) can impact the expression and function of ion channels and inhibiting outward potassium currents, which normally repolarize the cardiac cell after an action potential, can enhance inward depolarizing currents, such as the L-type calcium current (I_CaL_) and the sodium current (I_Na_), leading to prolonged action potentials (Lazzerini et al., 2023). The prolonged action potentials and spontaneous calcium release (as a result of phosphorylation, oxidation or change in expression of ryanodine receptor-2 (RyR2), calmodulin-dependent protein kinase II (CAMKII), sarcoplasmic reticulum calcium—adenosine triphosphatase–2a (SERCA2a) or phospholamban (PLB)) can lead to triggered activity, increasing the risk of arrhythmias (Lazzerini et al., 2023). Cytokines can affect gap junctions (formed by connexins, e.g. Cx-43), slowing and altering the homogeneous propagation of electrical impulses (Lazzerini et al., 2021; 2023). In more detail, IL-1 promotes atrial fibrosis and the modification of ion channel expression; IL-6 levels are commonly found in patients with atrial fibrillation, and this cytokine is believed to influence electrical conduction by altering gap junctions and intercellular communication; TNF-α can induce the release of reactive oxygen species (ROS), causes oxidative stress and ion channel dysfunction (Chen et al., 2020).

One of the most important procedures in patients with HFrEF and left ventricular ejection fraction (LVEF) ≤35% is device therapy with an implantable cardioverter-defibrillator (ICD) or cardiac resynchronization therapy with defibrillator (CRT-D), which reduce the risk of sudden cardiac death (SCD) (Breitenstein and Steffel, 2019; McDonagh et al., 2022; Naraen et al., 2023). The impact of inflammatory cytokines on arrhythmia triggering and high-energy therapies in patients with ICD/CRT-D is not fully understood. Higher cytokine level, especially interleukin 6 (IL-6) independently increased the risk of ICD shocks and is a predictor of SCD, however, data from the period of the COVID-19 pandemic (a systemic inflammatory disease) are not clear (Adabag et al., 2021; Cheng et al., 2014; Jagielski et al., 2018; Lazzerini et al., 2022; Streitner et al., 2009).

Majority of the available data concentrates on tumor necrosis factor-alpha (TNF-α), interleukin 1β (IL-1β), interleukin 6 (IL-6) and much less about interleukin 17 (IL-17) and inflammasomes (e.g. NLRP3). TNF-α, IL-1β, IL-6, IL-17 are key pro-inflammatory cytokines and are usually associated with an increased arrhythmic risk. The role of anti-inflammatory cytokine–IL-10 in ventricular arrhythmogenesis is ambiguous (Kondo et al., 2018; Lazzerini et al., 2022).

The inflammatory process also involves the NLRP3 inflammasome, a multi-protein signaling complex activated in response to damage associated molecular patterns (DAMPs) from dying cardiomyocytes. The NLRP3 inflammasome induces the production of potent pro-inflammatory cytokines such as IL-1β and IL-18 and is therefore another critical component of myocardial cell inflammation (Suetomi et al., 2018; Toldo and Abbate, 2018). In HF NLRP3 can increase cardiac myocyte hypertrophy, cardiac remodeling, myocardial fibrosis or inflammatory mediators (Wang et al., 2023). Moreover, inhibition of NLRP3 inflammasome slowed the HF process in animal model, can reduce myocardial fibrosis, delay the development of HF after myocardial infarction and the knockout of NLRP3 may reduce macrophage accumulation, which attenuated the inflammatory response and the development of fibrosis during HF (Wang et al., 2023). NLRP3 also modulates ROS production from the mitochondria to augment Smad signaling and fibrotic gene expression. This is the novel role for NLRP3 in the development of cardiac fibrosis through its interaction with members of the cellular redox machinery and involvement in cardiac myofibroblast differentiation (Bracey et al., 2014). Limited data are available about the NLRP3 inflammasome signaling and VAs. It seems, that inhibition of NLRP3 activation may reduce the incidence of VAs by reversing cardiac structural remodeling and electrical remodeling (Jiang et al., 2022; L. Zhang et al., 2022).

Klotho protein may be an extremely important cardioprotective factor between cytokine function, NLRP3, and arrhythmias. It can both suppress the production of pro-inflammatory cytokines, stimulate the production of anti-inflammatory factors, may influence calcium handling, fibroblasts activity and is involved in structural remodeling (Hung et al., 2022; Olejnik et al., 2022; Vázquez-Sánchez et al., 2025). The reduction in soluble Klotho may be associated with a pro-inflammatory status marked by lower IL-10 concentrations and higher TNF-α/IL-10 ratio and CRP levels (Martín-Núñez et al., 2020). Moreover, Klotho inhibits NLRP3 inflammasome activation and expression of TNF-α and IL-1β (Li et al., 2022).

In our recent study, we demonstrated significant redox disturbances in patients with HF and ICD/CRT-D interventions. We have proven that oxidative stress may be an additional risk factor for the development of arrhythmia in patients with HF (Szyller et al., 2023). There are no studies that clearly indicate the relationship between the inflammatory processes and ICD/CRT-D intervention or show possible changes in inflammatory parameters immediately after high-energy intervention. Therefore, in the current study, we aimed to characterize the levels of selected pro- and anti-inflammatory cytokines, soluble α-Klotho protein and complete blood count (CBC) in patients with HF and adequate high-voltage therapy with ICD/CRT-D as a result of VAs, to determine their possible impact on the occurrence of device shocks focusing on, among others, the NLRP3 inflammasome and the Klotho protein, which are currently one of the most intensively studied target in molecular cardiology.

## 2 Materials and methods

### 2.1 Participants

The study population consisted of 50 patients with diagnosed HF and implanted ICD or CRT-D devices. The sample size was adjusted to the practical possibilities of recruiting patients after high-voltage intervention in our center within 12 months. The inclusion and exclusion criteria are clearly defined in Table 1. All patients understood their participation in the research study and recognized its purpose. Written informed consent was obtained from all participants. The study was approved by the Bioethical Committee (KB 212/2023 and KB 142/2023) and is in compliance with the Helsinki Declaration. The general characteristics of the groups are presented in Table 2 which includes also the medications taken by the patients to assess their impact on the measured parameters. Both groups did not differ significantly in terms of the frequency of medication use.

**TABLE 1:** Detailed inclusion and exclusion criteria in the study.

Inclusion criteria		Exclusion criteria
Intervention group	Control group (ERI)	
1. Written consent of the patient to participate in the study 2. Diagnosed HF		1. No possibility of obtaining written consent for the study 2. Acute HF decompensation 3. The presence of comorbidities such as thyroid disease, cancer, HIV infection, viral hepatitis, valvular disease 4. Active inflammation or infection (e.g. myocarditis, pneumonia, pancreatitis, recent tooth extraction and dental treatment) 5. Smoking (including e-cigarettes) 6. Alcohol abuse (acceptable occasional consumption), use of psychoactive substances or stimulants
3. Registered ventricular arrhythmia and ICD/CRT-D shock (adequate intervention) 4. Absence of acute inflammation confirmed by CRP concentration measurement (serum CRP <15 mg/L) 5. No clinical features of HF decompensation	3. Qualification for ICD/CRT-D device replacement (positive Elective replacement indicator (ERI) status) 4. No ICD intervention min in the previous 3 months	

**TABLE 2:** General characteristics of the study groups.

	Intervention group	Control group	Intervention vs. control, *P-value*
Number of patients	21	29	
Age (years)	63.4 ± 2.7 (median: 70.0)	72.3 ± 1.6 (median: 72.0)	0.0214
Sex (male/female)	18/3	25/4	0.7164
Etiology of heart failure			
Ischemic	52% (n = 11)	45% (n = 13)	0.8096
Non-ischemic	48% (n = 10)	55% (n = 16)	
LVEF (%)	39.5 ± 10.8	36.9 ± 13.5	0.6245
NYHA			
I	48% (n = 10)	24% (n = 7)	
II	43% (n = 9)	59% (n = 17)	0.1534
III	9% (n = 2)	17% (n = 5)	0.4154
IV	---	---	0.7164
Arrhythmia			
VT	48% (n = 10)	---	---
VF	29% (n = 6)		
ES	23% (n = 5)		
Laboratory tests			
Na (mmol/L)	139.5 ± 2.2	141.6 ± 2.6	0.0038
K (mmol/L)	4.1 ± 0.4	4.2 ± 0.4	0.2038
Mg (mmol/L)	0.88 ± 0.09	0.87 ± 0.08	0.7761
TSH (µIU/mL)	2.507 ± 1.811	2.079 ± 0.888	0.2874
Creatinine (mg/dL)	1.11 ± 0.36	1.10 ± 0.32	0.8841
CRP (mg/L)	3.92 ± 4.66	2.52 ± 2.22	0.1676
Glucose (mg/dL)	122.8 ± 39.0	115.6 ± 25.3	0.4529
Comorbidities			
Hypertension	71% (n = 15)	76% (n = 22)	0.9792
Diabetes mellitus	23% (n = 5)	52% (n = 15)	0.0899
Hypercholesterolemia	67% (n = 14)	72% (n = 21)	0.9005
Classes of drugs			
ACEi	62% (n = 13)	59% (n = 17)	0.9534
Diuretics	67% (n = 14)	79% (n = 23)	0.4969
Beta-blockers	81% (n = 17)	93% (n = 27)	0.3875
Statins	67% (n = 14)	86% (n = 25)	0.1935
ARB	19% (n = 4)	31% (n = 9)	0.5306
Gliflosins	29% (n = 6)	38% (n = 11)	0.6987
Acetylsalicylic acid	23% (n = 5)	28% (n = 8)	0.9792
VKA	62% (n = 13)	34% (n = 10)	0.1025
Clopidogrel	4% (n = 1)	0% (n = 0)	0.8699

### 2.2 Blood collection

Blood samples from all patients were drawn from a median cubital vein or cephalic vein up to max. 6 h after appropriate discharge of ICD/CRT-D (“intervention group”), or from patients qualified for ICD/CRT-D device replacement (positive Elective Replacement Indicator (ERI) status) and with no high-voltage intervention delivered in the past 3 months (“control group”) after admission to hospital during routine tests to a tube with a clot activator (VACUETTE® TUBE 4 mL CAT Serum Clot Activator, Greiner Bio-one Cat. no. 454092) to obtain serum. Blood samples were transported from cardiology unit to the clinical laboratory in a cooler within max. 20 min of being drawn, then centrifuged at 2,000 x g for 10 min 300 μL of serum has been transferred to the polypropylene microtubes (Eppendorf, Hamburg, Germany) and stored until use at −80 °C.

### 2.3 Determination of TNF-α and IL-6 concentration

Commercial enzyme-linked immunosorbent assays (ELISA) test kits: Human IL-6 DuoSet ELISA (Cat. No. DY206) and Human TNF-alpha DuoSet ELISA (Cat. No. DY210) (R&D Systems, Inc., Minnesota, MN, USA), were used in accordance with manufacturer’s protocols. IL-6/TNF-α was immobilized by mouse anti-human IL-6 capture antibody/mouse anti-human TNF-α capture antibody and was detected using biotinylated goat anti-human IL-6 detection antibody/biotinylated goat anti-human TNF detection antibody along with streptavidin conjugated to HRP. In each assay, the reaction was developed using a 3,3′,5,5′-Tetramethylbenzidine (TMB) substrate solution. The substrate reaction was stopped, and the extinction was measured at 450 nm with the correction read at 540 nm using an ELISA reader. The kits provided standards ranging from 0 pg/mL to 600 pg/mL for IL-6 and 0 pg/mL to 1,000 pg/mL for TNF-α. If the measurements were out of the range, the samples were diluted, and the concentration read from the standard curve was multiplied by the dilution factor. We performed the analysis in the double-check mode, which means we measured each sample twice to reduce the likelihood of random errors. The ELISA test reproducibility can be assessed through intra-assay (within-run) and inter-assay (between-run) precision. Intra-assay precision for TNF-α and IL-6 were 4.2% and 6.5% respectively inter-assay precision were 2.4% and 6.4% respectively.

### 2.4 Determination of Il-1β, IL-10, high sensitive IL-17 and NLRP3 concentration

Interleukins and inflammasome NLRP3 concentration were measured using FineTest® ELISA Kits (Wuhan Fine Biotech Co., Ltd., China; Cat. No.: EH0185 (IL-1β), EH0173 (IL-10), AQ-H3267-B (HS-IL-17), EH4202 (NLRP3)). Each analysis was conducted in duplicate. The microplate has been pre-coated with a human anti-Il-1β, anti-IL-10, anti-IL-17 or anti-NLRP3 antibody. Samples were added to the wells and the biotin conjugated anti-IL-1β, anti-IL-10, anti-IL-17 or anti-NLRP3 antibody were used as the detection antibody. After washing off the wells, horseradish peroxidase (HRP) with streptavidin and then TMB was added to visualize HRP enzymatic reaction. TMB was catalyzed by HRP to produce a color product. The color change was measured spectrofotometrically at a wavelength of 450 nm (Spark Multimode Microplate Reader, Tecan Trading AG, Switzerland). The concentration of IL-1β, IL-10, IL-17 and NLRP3 in the samples is proportional to the OD450 value and were calculated by drawing a standard curve. Sensitivity of the tests was: 2.344 pg/mL for Il-1β, 4.688 pg/mL for IL-10, 3.75 pg/mL for HS-IL-17 and 0.469 ng/mL for NLRP3. The analysis was performed in the double-check mode. Intra-assay precision for IL-1β, IL-10, IL-17 and NLRP3 were max. 4.98%, 5.18% 5.23%, 5.15% respectively and the inter-assay precision were max. 4.72%, 5.29%, 5.55%, 5.22% respectively.

### 2.5 Determination of soluble α-Klotho protein and FGF-23

Soluble α-Klotho concentration was measured with a solid-phase sandwich Alpha Klotho Human Soluble ELISA Kit #JP27998 (IBL Co. Ltd., Minneapolis, MN, USA), according to the manufacturer’s instructions. The concentration of FGF23 was determined using Human FGF23 ELISA Kit #orb390902 (Biorbyt Ltd., Cambridge, United Kingdom). The color change after standard ELISA reaction (with the use of TMB) was measured spectrophotometrically at a wavelength of 450 nm (Spark Multimode Microplate Reader, Tecan Trading AG, Switzerland). To ensure the validity of the method, all measurements were performed in duplicate. Intra-assay precision for α-Klotho 23 was max. 3.5% and the inter-assay precision was 6.5%. Intra-assay inter-assay precision for FGF-23 was not provided by the manufacturer.

### 2.6 Complete blood count and CRP determination

Peripheral complete blood count (CBC, 5-diff) and CRP test were performed on Sysmex XN-1000 (Sysmex Corporation) analyzer (IVD) after the patient was admitted to the hospital - before ICD/CRT-D replacement (ERI group) or immediately after admission to hospital due to high-voltage therapy. The results used came from the patient’s electronic records. In ERI group only 18 patients had CBC 5-diff.

### 2.7 Statistical analysis

The experimental data were analyzed using GraphPad Prism 8.0.1 for Windows (GraphPad Software, San Diego, California, USA). We used the Shapiro-Wilk normality test to assess the normality of a dataset. For comparisons between two independent groups of measurements of normally distributed data the Student’s t-test was used. The data without normal distribution were compared with the Mann-Whitney U test. In the case of unequal variances, the t-test with Welch correction was used. Yates’s Chi-squared test was used to compare categorical variables between the groups. Results were expressed as mean ± standard error of the mean (SEM) or as median with interquartile range (IQR) according to data distribution. P value of <0.05 was regarded as statistically significant.

## 3 Results

**FIGURE 1:**
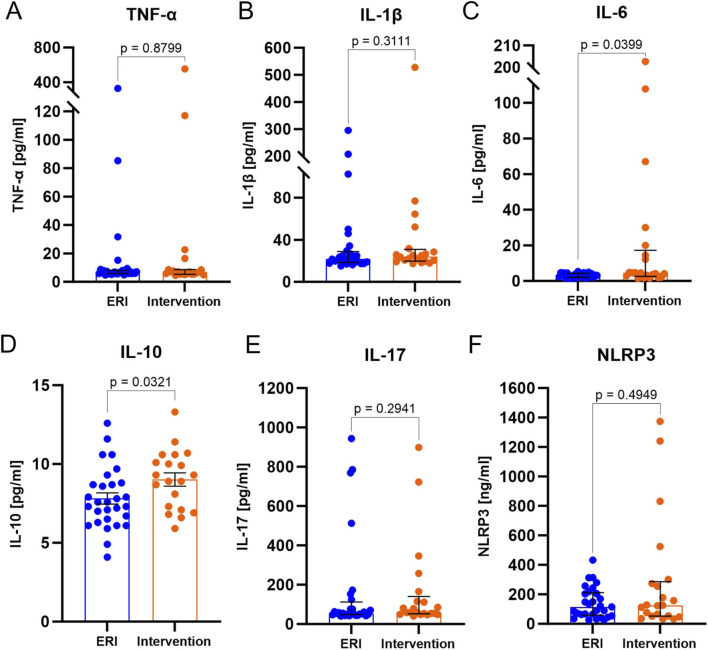
Cytokine and NLRP3 inflammasome serum concentration in ERI and intervention group. Tumor necrosis factor α, TNF- α **(A)**, interleukin 1β, IL-1β **(B)**, interleukin 6, IL-6 **(C)**, interleukin 10, IL-10 **(D)**, interleukin 17, IL-17 **(E)**, NLR family pyrin domain containing 3, NLRP3 **(F)**. Data were presented on a box-plot as median with interquartile range, except for IL-10, where data was presented as mean ± SEM (normal distribution). P-value were provided for each parameter. ERI group, n = 26; Intervention group, n = 21.

The serum concentration of TNF-α, median 6.5 (95% CI: 5.8–7.3) vs. 6.8 (95% CI: 5.4–8.5) pg/mL, p = 0.8799, Figure 1A, IL-1β median 21.0 (95% CI: 19.1–24.7) vs. 24.3 (95% CI: 20.5–28.4) pg/mL, p = 0.3111, Figure 1B, IL-17 median 54.9 (95% CI: 49.9–76.3) vs. 59.1 (95% CI: 54.5–115.0) pg/mL, p = 0.2941, Figure 1E) and NLRP3 median 115.4 (95% CI: 65.5–202.8) vs. 126.2 (95% CI: 53.8–272.0) ng/mL, p = 0.4949, Figure 1F) did not differ between ERI and intervention groups. IL-6 was higher in the group after ICD/CRT-D intervention (3.3 pg/mL, 95% CI: 2.7–3.7 vs. 4.6 pg/mL, 95% CI: 2.9–14.5, p = 0.0399, Figure 1C). In this group, IL-10 reaches also significantly higher concentrations (mean 7.8 pg/mL, 95% CI: 7.1–8.5 vs. 9.0 pg/mL, 95% CI: 8.1–9.9, p = 0.0321, Figure 1D). The results indicate possible role of cytokines in VAs. High TNF-α concentrations achieved in several patients coincided with high IL-1β and IL-6 values, which clearly indicates the stimulation of a pro-inflammatory response.

**FIGURE 2:**
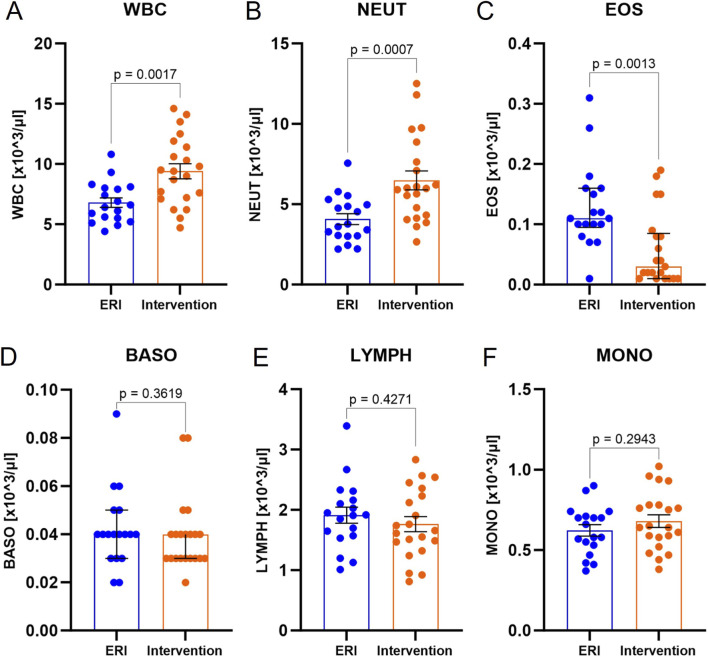
Peripheral 5-diff blood count in ERI and intervention group. Total white blood cells, WBC **(A)**, neutrophils, NEUT **(B)**, eosinophils, EOS **(C)**, basophils, BASO **(D)**, lymphocytes, LYMPH **(E)**, monocytes, MONO **(F)**. Data were presented on a box-plot as mean ± SEM except for EOS and BASO, where data was presented as median with interquartile range. P-value were provided for each parameter. ERI group, n = 18; Intervention group, n = 21.

Importantly, the group after ICD/CRT-D intervention was characterized by a higher number of white blood cells (WBC) in peripheral blood (6.8 × 10^3^/µL, 95% CI: 6.0–7.6 vs. 9.4 × 10^3^/µL, 95% CI: 8.1–10.7, p = 0.0017, Figure 2A) without a significant difference in C-reactive protein (CRP) concentration (median 1.7 mg/L, 95% CI: 1.1–3.0 vs. 2.6 mg/L, 95% CI: 1.1–6.1, p = 0.2893). The increase in WBC count was due to the increased number of neutrophils (NEUTs) (4.1 × 10^3^/µL, 95% CI: 3.4–4.8 vs. 6.5 × 10^3^/µL, 95% CI: 5.3–7.7, p = 0.0007, Figure 2B). A reduced eosinophils (EOSs) count was also observed (0.11 × 10^3^/µL, 95% CI: 0.10–0.16 vs. 0.03 × 10^3^/µL, 95% CI: 0.01–0.08, p = 0.0013, Figure 2C). Other CBC 5-diff morphology parameters did not differ between ERI and intervention group. Increased neutrophils and decreased eosinophils during inflammation, especially in severe cases (e.g. during COVID-19), is a common observation.

**FIGURE 3:**
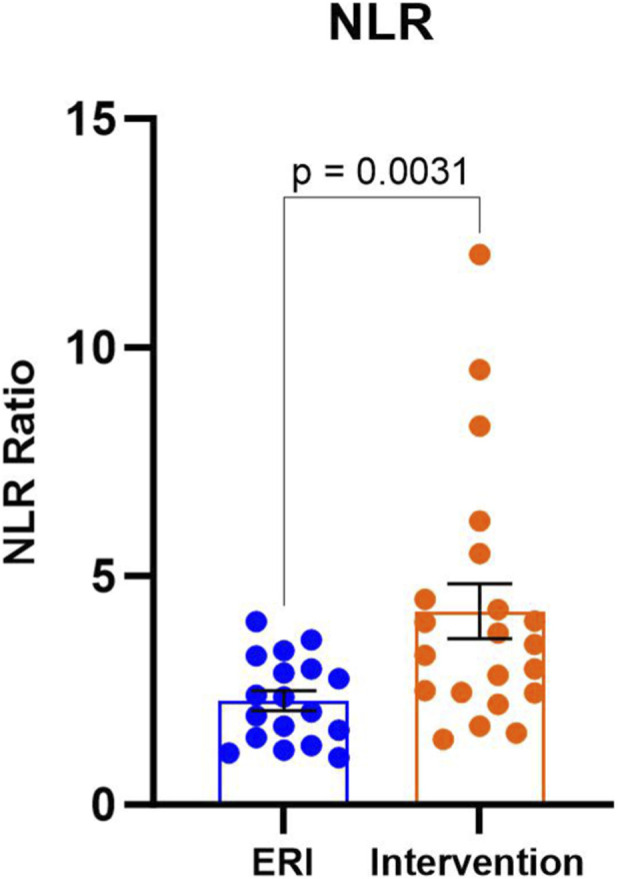
Neutrophil-to-lymphocyte (NLR) ratio in ERI and intervention group. Data were presented on a box-plot as median with interquartile range. ERI group, n = 18; Intervention group, n = 21.

The neutrophil-to-lymphocyte (NLR) ratio was also increased in patients after ICD/CRT-D intervention (2.2, 95% CI: 1.5–3.0 vs. 3.5, 95% CI: 2.5–4.5), p = 0.0031, Figure 3). Elevated NLR suggests a shift towards a pro-inflammatory state, as neutrophils are involved in the initial inflammatory response.

**FIGURE 4:**
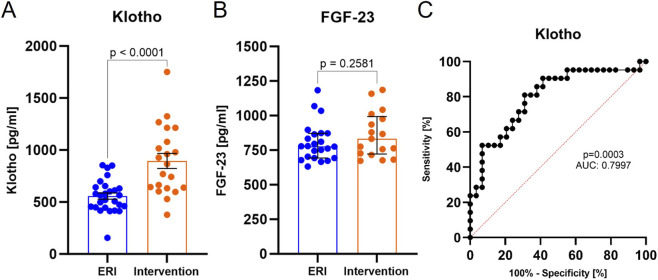
Human soluble α-Klotho protein **(A)**, FGF-23 **(B)** serum concentration in ERI and intervention group and **(C)** ROC curve for Klotho protein. Data were presented on a box-plot as mean ± SEM for soluble Klotho protein and as median with interquartile range for FGF-23. P-value were provided for each parameter. ERI group, n = 26; Intervention group, n = 21.

**FIGURE 5:**
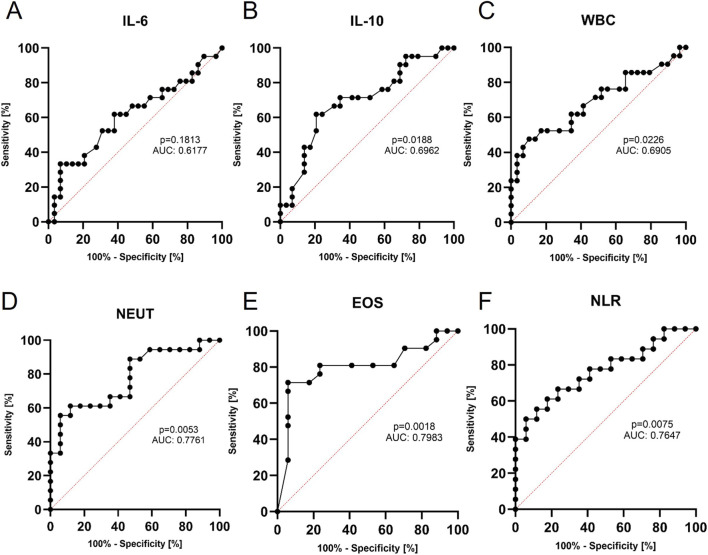
ROC curves for cytokines: IL-6 **(A)**, IL-10 **(B)** and for important blood count parameters: WBC **(C)**, NEUT **(D)**, EOS **(E)** and neutrophil-to-lymphocyte ratio **(F)**. AUC and p-value were provided for each potentially significant parameter. The most diagnostically useful indicator for device intervention seems to be eosinophil count with sensitivity of 81.0% and specificity of 76.5% (AUC 0.7983, 95% CI: 0,6465–0.9502, p = 0.0018) with a cut-off value of <0.09 × 10^3^/µL).

Serum concentration of soluble Klotho protein was significantly higher after device intervention (557.5 pg/mL, 95% CI: 495.5–619.5 vs. 895.2 pg/mL, 95% CI: 744.7–1,046.0, p < 0.0001, Figure 4A), with no change in FGF-23 levels (778.0 pg/mL, 95% CI: 703.0–864.1 vs. 831.4 pg/mL, 95% CI: 726.8–975.9), p = 0.2581, Figure 4B). Klotho was characterized by sensitivity of 85.7% and specificity of 62.1% for cut-off concentration >602.7 pg/mL in the peri-shock period (Figure 4C). This indicates significant induction of Klotho in the peri-shock period. This may indicate the clinical usefulness of measuring Klotho levels to determine the cut-off value of arrhythmia risk in patients with HF or to assess the inflammatory/oxidative stress response following ICD/CRT-D high voltage intervention. However, this requires further detailed clinical trials. The ROC for the remaining selected parameters is presented in Figure 5.

## 4 Discussion

A role for inflammation in HF has been proposed for decades. Multiple studies have demonstrated the potential involvement various cytokines and chemokines in acute and chronic HF, which is associated with, among other, oxidative stress or pro-fibrotic mechanisms. The imbalance between pro- and anti-inflammatory cytokines constitutes the pathophysiological basis of HF and may be a risk factor for VAs (Boulet et al., 2024).

Our study shows that IL-6 and IL-10 level were significantly higher after ICD/CRT-D intervention. Increased IL-6 level in intervention group may be due to short-time VF/VT and injury of myocardium caused by ICD/CRT-D discharge (Stieger et al., 2018). In some studies, elevated levels of IL-6 and IL-10, along with other inflammatory cytokines have been linked to a higher risk of VAs. IL-6 may also promote VF/VT by increasing QT duration (increased density of I_CaL_ and suppressed I_Kr_) (M. Chen et al., 2020). Moreover, ICD patients with initially elevated IL-6 levels have a significantly higher risk of appropriate ICD shocks (Cheng et al., 2014). Short-term action of IL-6 shows cardioprotective influence by propagating myocardium regeneration after acute damage but with long-term elevation has pathogenic effect, causing fibrosis and chronic inflammation (Fontes et al., 2015). Elevated IL-6 level in patients recently hospitalized due to HF is related to increased risk of death (all-cause as well as cardiovascular) and re-hospitalization because of HF but may not for VF/VT incident rate (Sourour et al., 2024).

IL-10 is known as an anti-inflammatory cytokine. Increased IL-10 level in our study is related with IL-6 and Klotho protein and may be a response to inflammation. IL-10 has a potential protective function but is also a marker of inflammation intensity. Studies in animal models have shown that administration of IL-10 reduced fibrosis and improved cardiac electrical parameters (Jung et al., 2017) and IL-10 overexpression may decrease VAs occurrence rate. Further studies are required to determine whether IL-10 has a modulating function in arrhythmias.

TNF-α, IL-1β, IL-17 and NLRP3 levels were similar in both groups. TNF-α is the most studied cytokine in terms of HF development and its effect on VF/VT. Increased TNF-α due to myocardium injury is responsible for increased ROS concentration by decreasing activity of complex-III and level of mtDNA (Suematsu et al., 2003). Moreover, TNF-α affects the electrophysiological properties of cardiomyocytes by decreasing the transient outward K^+^ current (I_to_) and elongation of action potential duration (APD) (Fernández-Velasco et al., 2007). Also, Cx-43 expression is decreased by TNF-α (Della Morte et al., 2022) which results in higher risk of reentry and induction of VF/VT. Thus TNF-α were suspected of HF development and increased rate of VF/VT.

NLRP3 inflammasome is crucial in IL-1β activation. NLRP3 and IL-1β level may vary due to early short activation of NLRP3 or not yet produced IL-1β. Elevated IL-1β disturb Ca^2+^ metabolism and leads to APD elongation (M. Chen et al., 2020). There are no publications comparing NLRP3 levels in patients after ICD/CRT-D shock. In our study serum NLRP3 and IL-1β concentration did not differ between groups. This may be due to local activation of NLRP3 and synthesis of IL-1β, which does not affect the level in peripheral blood (Heger et al., 2024). However, local activation of NLRP3 in myocardium may be crucial for arrhythmogenesis but this requires further research.

We also checked IL-17 level as a marker of HF severity and intensity of inflammatory processes (Baumhove et al., 2024). Our studies show no difference in IL-17 levels between groups. Increased IL-17 concentration may involve long-term remodeling rather than acute reaction and may be important in VAs occurrence rate. IL-17 shows a proarrhythmic effect by increasing APD and decreasing conduction velocity at the cardiomyocyte membrane level and is responsible for heart fibrosis (Li et al., 2022). In animal model IL-17 leads to adverse postmyocardial infarction remodeling by inducing cardiomyocyte apoptosis. IL-17 may also promote neutrophil infiltration and cardiac fibrosis by upregulating the expression of lncRNA-AK081284 and transforming growth factor β1 (TGF-β1) (Li et al., 2022; Zhang et al., 2018). Knockout of IL-17 alleviated interstitial fibrosis as manifested by reduced mRNA expression of collagen type I, collagen type III, α-smooth muscle actin, TGF-β1 and collagen deposition, which can be used as markers of fibrosis (Zhang et al., 2018). It would be valuable to conduct a study examining the level of IL-17 and fibrosis markers to assess whether elevated IL-17 may have an arrhythmogenic effect. There are also no studies on humans that would show the relationship between IL-17 levels and the occurrence of VF/VT.

Klotho, a transmembrane and secretory protein, is primarily produced in the kidneys and brain, as well as in the heart and other tissues. It is a co-receptor for fibroblast growth factor 23 (FGF23) (Olejnik et al., 2020) and has antioxidant, anti-inflammatory and antifibrotic properties (Olejnik et al., 2018). The protective role of Klotho in renal disorders has been shown, where Klotho deficiency acted as an early biomarker and therapeutic target in acute kidney injury (AKI) and chronic kidney disease (CKD) (Grange et al., 2020; Liu et al., 2019). Crucially, abnormal levels and activity of Klotho and FGF23 are highly related to cardiovascular disease (CVD) (Donate-Correa et al., 2013). In our study, Klotho level was higher in patients with HF after appropriate ICD/CRT-D intervention. This increase can indicate a physiological reaction to treatment to prevent pro-inflammatory response and VAs, driven by enhanced *de novo* Klotho synthesis and representing a compensatory mechanism that facilitates cardiac repair. Alternatively, it may arise from Klotho release by stressed or damaged cells as a consequence of cellular injury processes. It has been reported that patients with HF exhibited elevated circulating Klotho levels, particularly those who demonstrated clinical improvement following intensive treatment. Upon recovery, Klotho level declined to baseline values, and was proposed as a potential novel biomarker for monitoring treatment responsiveness (Taneike et al., 2021). The compensatory synthesis of Klotho was also found in patients after myocardial infarction (Olejnik et al., 2025). The role of Klotho in inflammation is linked with the regulation of inflammatory factors expression, as well as the interference with the phenotype and function of inflammatory cells like monocytes, macrophages, T cells, and B cells (Zhao et al., 2024). It has been shown that Klotho exerts anti-inflammatory effects by suppressing NF-κB signalling and NLRP3 inflammasome activation, leading to reduced production of pro-inflammatory cytokines such as IL-6 and TNF-α (Fu et al., 2023). The immunomodulatory and cardioprotective effect of Klotho was also indicated by Junho et al. (2022), where Klotho protected against low cell contraction, loss in the systolic Ca^2+^ transients, sarco/endoplasmic reticulum Ca^2+^-ATPase (SERCA2a) activity and the occurrence of pro-arrhythmic events (Junho et al., 2022). Importantly, Klotho supplementation mitigated functional and structural cardiac remodelling and reduced ventricular arrhythmic events in mice post-myocardial infarction by preventing dysregulation of intracellular Ca^2+^ handling (Vázquez-Sánchez et al., 2025). In hemodialysis patients or those undergoing catheter-based percutaneous radiofrequency, Klotho was shown to be protective against atrial fibrillation (AF) (Mizia-Stec et al., 2018; Nowak et al., 2014). To sum up, elevated Klotho levels may indicate a compensatory reaction that mitigates inflammation and arrhythmias, serving as a potential marker of therapeutic response in HF. Methods for measuring Klotho concentration are not standardized. The mean Klotho serum concentration in the United States (US) population (40–79 years old) according to the National Health and Nutrition Examination Survey (NHANES) database is 849.33 ± 5.39 pg/mL (measured using an ELISA kit, IBL International; the same as in our study) and is closely related to Life’s Essential 8 components (Kadier et al., 2024). Decreased Klotho concentration is associated with a higher risk of HF and is confirmed by our observations in ERI group (patients with HF) and in CHF patients from the Frankfurt Bone Marrow-Derived Cell Therapy Registry which had a lower serum Klotho concentration than controls (the median serum Klotho concentration was 674 pg/mL in CHF patients and 903 pg/mL in controls; IBL International ELISA kit) (von Jeinsen et al., 2019). However, there is a lack of data to determine cut-off values ​​for clinical decisions related to HF and VAs.

The significant revelation is that cardiac leukocytes play a critical role in maintaining normal cardiac electrical conditions. They may be directly involved in the pathophysiological mechanisms underlying arrhythmias (Hegemann et al., 2024). Circulating leukocytes are crude markers of the systemic immunological status, and they can modulate local inflammatory responses (Chen et al., 2023). One of the subpopulations–NEUTs, constitute an innate immune system and represent 50%–70% of all human leukocytes (Summers et al., 2010). They are typically the first responders to inflammation and can both contribute to tissue damage and promote tissue repair. Their involvement in arrhythmias may be related to their ability to promote inflammation in the heart tissue, which can disrupt normal electrical activity and electrical propagation through the conduction system. Tissue NEUTs carry out proinflammatory effector functions and are detected in the heart muscle under physiological conditions. The key mechanism for NEUTs’s proarrhythmogenic action is neutrophil-mediated oxidative stress generation via lipocalin-2 (Lcn2) pathway (Grune et al., 2022). High levels of NEUTs and low levels of EOSs, which are observed in our study, are associated with an increased risk of certain types of arrhythmias, including VAs and AF (Chen et al., 2023; Hegemann et al., 2024). EOSs, also involved in inflammation, can modulate immune responses and potentially contribute to cardiac remodeling. Their role in arrhythmias is less clear than that of NEUTs, but they may have protective effects against certain types of heart rhythm problems. Not all studies confirm these observations, except for NEUTs and LYMPHs (Chen et al., 2023).

EOSs count were independent predictors of AF recurrence during antiarrhythmic drug therapy (OR: 1.643 per 1 × 10^8^/L increase; 95% CI: 1.047–2.578; P = 0.031) (Chen et al., 2017), however their association with VAs is not clear, except in eosinophilic myocarditis (EM). In EM here they may be related to a recurrent electrical storm consisting of multiple episodes of sustained ventricular tachycardia (Hengst et al., 2024). IL-10 is described as an inhibitor of eosinophilia by suppressing the production of IL-5 and GM-CSF, indirectly influencing the apoptosis and proliferation of eosinophils (Akdis and Blaser, 2001). This is fully consistent with our observations in patients after high-voltage therapy. They had a 58% higher number of NEUTs and a 72% lower number of EOSs. Moreover, IL-10 level is significantly associated with Klotho. Studies show that patients with cardiovascular disease, including HF, often have lower serum levels of both soluble Klotho and IL-10 and the higher values of serum Klotho is associated with decreased values of the TNF-α/IL-10 ratio (Martín-Núñez et al., 2020). In NHANES study in US adults higher α-Klotho was associated with lower eosinophils and with lower inflammatory markers (e.g. neutrophils and CRP) (Han et al., 2023). The upregulation of IL-10 results from Klotho-mediated activation of the JAK2/STAT3 signaling axis (Typiak and Piwkowska, 2021). This confirms our observations in this study. Klotho may also reduce the expression of pro-inflammatory factors and may be important in the Klotho-IL-10-eosinophils pathway in patients with cardiovascular diseases. This observation may be very important in patients with hypereosinophilic syndrome (HES) associated with arrhythmias.

In the future, the suppression of the NLRP3 inflammasome and its signaling path is expected to provide a new intervention mediator for the therapy of HF. Right now many studies have presented that therapy with inhibition of pro-inflammatory cytokines such as IL-1 or IL-18 has promise as a new direction for the treatment of HF and may cause increased oxygen consumption, may improve exercise tolerance and may reduce cardiac fibrosis (Buckley et al., 2018; Mallat et al., 2004; van Tassell et al., 2012). NLRP3 inflammasome has been also proposed as a potential intervention mediator to treat multiple inflammatory diseases. In many studies, the use of NLRP3 inhibitors resulted in a reduction in fibrosis, improvement in systolic and diastolic heart function, increased LVEF and a decrease in hospitalization rate (Wang et al., 2023; Wohlford et al., 2020). We believe that this clinical approach may also reduce the frequency of Vas and ICD/CRT-D intervention in patients with HF. In studies directly related to heart rhythm disorders, anti-inflammatory therapies, cytokine inhibitors, and immunomodulatory agents show promise in reducing inflammation-driven supraventricular and ventricular arrhythmias (Obeagu, 2025). Also, the possibility of using the Klotho protein as an antiarrhythmic agent may be extremely interesting. Klotho may protect against QT prolongation (Navarro-García et al., 2022). Klotho supplementation also protected against functional and structural cardiac remodelling and ameliorated ventricular arrhythmic events by preventing intracardiomyocyte Ca^2+^ mishandling (Vázquez-Sánchez et al., 2025).

## 5 Conclusion

Inflammation is a major pathophysiological contributor of HF and inflammatory activation is increasingly recognized as a risk factor for cardiac arrhythmias that trigger ICD/CRT-D shocks. This can manifest as direct effects on the heart as: prolonging the action potential duration, impacting calcium handling within heart cells, causing structural changes, like cardiac fibrosis, that further increase arrhythmia risk or indirect, e.g. overactivation of the cardiac sympathetic nervous system. In our study IL-6 and IL-10 level were higher after ICD/CRT-D intervention and these patients have a significantly higher risk of appropriate ICD shocks. In this group of patients the observation is also confirmed that high levels of NEUTs and low levels of EOSs may be associated with an increased risk of VAs. The use of the Klotho protein in diagnostics seems to be extremely interesting as it may have a significant preventive antioxidative and anti-inflammatory effects. Further research and clinical trials with particular emphasis on IL-6, IL-10, blood count and Klotho protein may be useful in assessing the risk of ICD/CRT-D intervention.

## 6 Limitations

Our study had several important limitations and its results should be interpreted critically. One of the most important limitations was the small size of the study groups, a single-center study design and lack of longitudinal data. This was due to organizational and logistical difficulties. However, the current results indicate the need for more detailed research, which will be conducted at our center.

## Data Availability

The raw data supporting the conclusions of this article will be made available by the authors, without undue reservation.
